# Clinical and economic consequences of medication nonadherence: a review of systematic reviews

**DOI:** 10.3389/fphar.2025.1570359

**Published:** 2025-06-25

**Authors:** Maria Achterbosch, Nilay Aksoy, George D. Obeng, David Ameyaw, Tamás Ágh, Job F. M. van Boven

**Affiliations:** 1 Department of Clinical Pharmacy & Pharmacology, University Medical Center Groningen, University of Groningen, Groningen, Netherlands; 2 Department of Clinical Pharmacy, School of Pharmacy, Altinbas University, Istanbul, Türkiye; 3 Syreon Research Institute, Budapest, Hungary; 4 Syreon Research Africa, Accra, Ghana; 5 Medication Adherence Research Group, Center for Health Technology Assessment and Pharmacoeconomic Research, University of Pécs, Pécs, Hungary; 6 Department of Clinical Pharmacy & Pharmacology, Medication Adherence Expertise Center of the Northern Netherlands (MAECON), University Medical Center Groningen, University of Groningen, Groningen, Netherlands

**Keywords:** medication adherence, economic outcomes, clinical impact, burden, cost-effectiveness, chronic diseases, adherence, clinical outcomes

## Abstract

**Background:**

Medication efficacy observed in clinical trials may differ from its effectiveness during real-world usage. Medication nonadherence is one of the key factors being responsible for this efficacy-effectiveness gap. The World Health Organization estimated that only 50% of chronic medication users is adherent and nonadherence results in both negative health outcomes for the patient and higher societal costs. An overview of the consequences across disease groups may allow some comparison and could contribute to identification of priority clinical areas.

**Objective:**

We aimed to provide an overview the impact of nonadherence on clinical and economic outcomes.

**Method:**

We narratively reviewed systematic reviews published between 2014 and 2024 on the effect of medication nonadherence on clinical and economic outcomes.

**Results:**

Overall, 43 systematic reviews were identified, including over 410 original studies on clinical outcomes and 174 on economic outcomes, covering different clinical areas (e.g., organ transplantation, cardiovascular diseases, diabetes, depression and chronic lung diseases [asthma/COPD]). Beyond diminished treatment effects, medication nonadherence has been associated with elevated mortality, increased healthcare utilization (including hospital admissions), and higher direct (e.g., more healthcare provider visits) and indirect financial cost burden (e.g., work productivity losses due to absenteeism and presenteeism) for patients and society.

**Conclusion:**

Medication nonadherence is associated with poor clinical and economic outcomes across disease areas. Given the significant impact of nonadherence, raising awareness among healthcare professionals and policymakers, early stakeholder engagement in intervention design, and eventually implementation of cost-effective interventions on both health policy, system and individual patient level are urgently required.

## Introduction

Medication is the cornerstone treatment prescribed for most chronic diseases such as asthma, diabetes and osteoporosis. Generally, these medications have been extensively evaluated in randomized clinical trials and have been granted market access based on a positive benefit-risk ratio. However, this positive benefit-risk ratio may not always be observed in daily real-world practice and one the key determining factors for this discrepancy is medication nonadherence.

Medication adherence has been defined by the World Health Organisation (WHO) as “the extent to which a person’s behaviour–taking medication, following a diet, and/or executing lifestyle changes, corresponds with agreed recommendations from a healthcare provider.” (1) Globally, the WHO estimated that around half of all chronic patients do not take their medicine according to prescription (World Health Organization, 2003). This is not without consequences. The Organization for Economic Co-operation and Development (OECD) estimates that medication nonadherence has been associated with 200,000 deaths and €125 billion avoidable medical expenditures per year in Europe in patients (Khan and Socha-Dietrich, 2018). In the USA, similar figures have been estimated with reported avoidable medical expenditures of $100–300 billion per year due to adverse drug events of which one-third was attributed to medication nonadherence (Cambridge, 2009; Senst et al., 2001). These negative consequences of medication nonadherence for patients have been shown in multiple studies as well. Already in 2002, a meta-analysis demonstrated the overall significant negative impact of therapy and medication nonadherence on treatment outcomes such as pain, risk of cardiovascular events and morbidity in a variety of disease areas (Robin DiMatteo et al., 2002). In the years that followed, multiple additional studies have been published confirming and extending these findings.

However, while overall estimates are essential to raise awareness and shape policy, most of the previous studies focused on the effect of adherence enhancing interventions (Kini and Ho, 2018), on the specific treatment outcomes (Robin DiMatteo et al., 2002), focused only on the economic outcomes (Cutler et al., 2018; Iuga and McGuire, 2014) or both on clinical and economic outcomes but in specific disease groups, e.g., COPD (van Boven et al., 2014), or populations, e.g., aging population (Walsh et al., 2019). More holistic insight into the clinical and economic impact of nonadherence per disease group may however be more informative for policymakers to inform the overall potential of these adherence supporting interventions in terms of cost-effectiveness, budget impact, scale-up and implementation.

We aimed to assess the clinical and economic impact of medication nonadherence by narratively reviewing previously published systematic reviews across chronic diseases.

## Materials and methods

### Study design

This semi-systematic narrative review was reported according to the Scale for the Assessment of Narrative Review Articles (Baethge et al., 2019).

### Search strategy and selection process

Two semi-structured searches were performed in Medline via PubMed using combinations of the following search terms: medication adherence AND burden, economic, impact, outcomes, clinical, AND systematic review. One search focused on the clinical impact of medication nonadherence and the other on the economic impact of medication nonadherence (see Supplementary Material for detailed search strings). The literature search was performed in May 2024. To provide an up-to-date overview, articles were filtered by publication date; only articles published after 1 January 2014 were screened. Reference lists of relevant articles were inspected to identify further relevant systematic reviews.

Study inclusion criteria were (1) the study design was a systematic review and/or meta-analysis, (2) the study assessed the relationship between nonadherence to medication and any clinical and/or economic outcomes in any disease area as main or secondary study outcome, and (3) the study was published in English.

All articles on clinical outcomes were screened for eligibility by one researcher (MA) on title and abstract. In case of doubt, the full-text article was screened or the article’s title and abstract were screened by a second researcher (NA). This same process was also performed concerning the articles on economic outcomes, but by two other researchers (GO and DA).

### Data items and extraction process

Data from relevant studies were extracted and checked independently by two researchers. Subsequently, data were manually tabulated in a Microsoft Excel file.

The following data items were extracted for each article: last name of first author and year of publication, the number of included original studies, the number of included original studies with a clinical or economic outcome, the clinical area or disease, type of medication, definition of adherence, the clinical or economic outcome, significance of the impact on the clinical or economic outcome (significant or non-significant), and direction of the relation between nonadherence and the clinical or economic outcome (positive or negative). In case of meta-analysis, the overall significance was extracted. In case of missing data or when data were described unclearly, this was reported as not reported.

Reported cost data in the included reviews were adjusted to 2024 US$ using the consumer price index for all urban consumers (CPI-U), applying January values to account for inflation across the specified period. As all relevant cost data in the included reviews were originally reported in USD, no currency exchange adjustments were necessary.

## Results

### Search results

**FIGURE 1:**
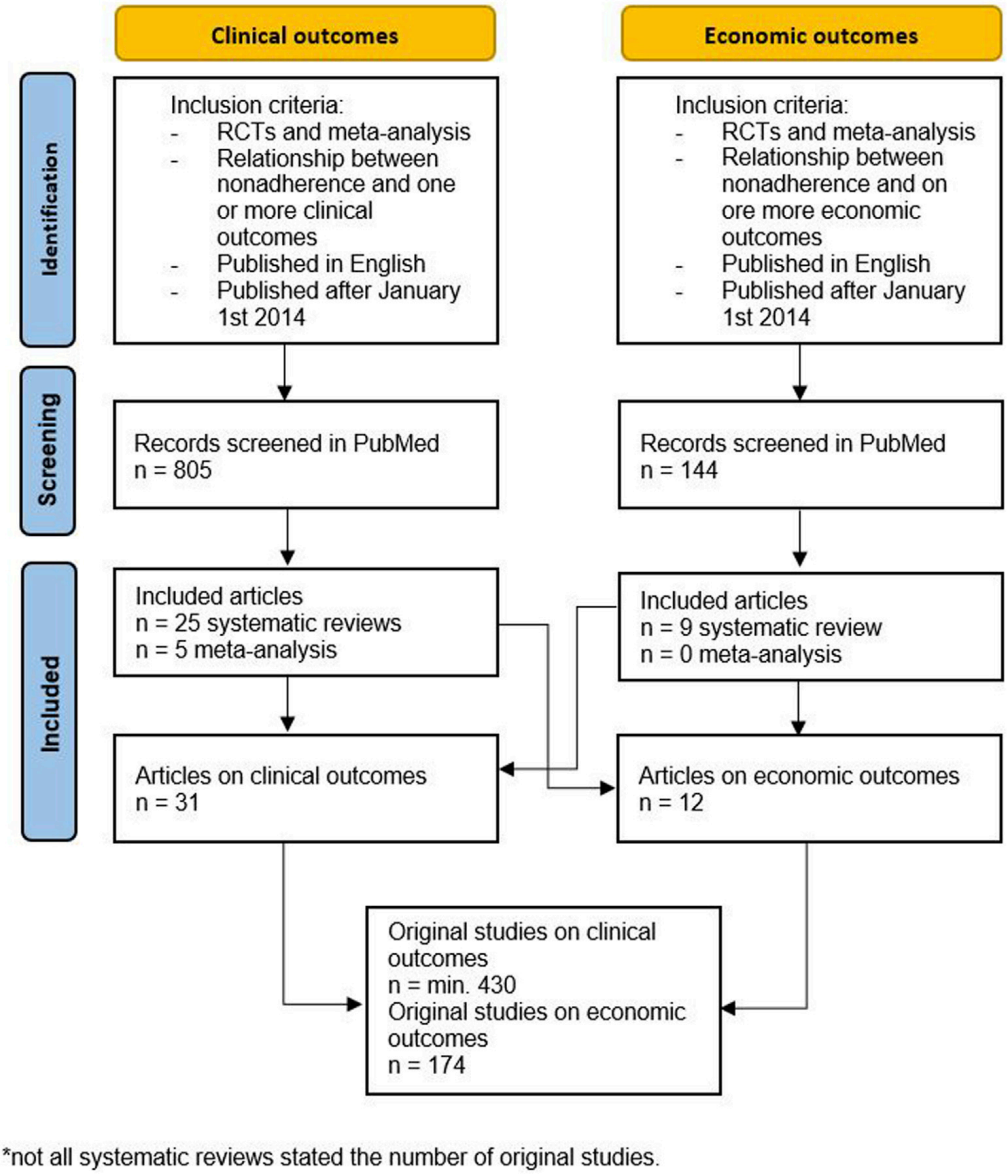
Flowchart of included articles.

In total, the searches yielded 43 relevant systematic reviews and meta-analyses (*N* = 5) (Walsh et al., 2019; Altice et al., 2019; Souza et al., 2016; Lee et al., 2022; Shehab et al., 2019). After inspection of reference lists, no more relevant studies were identified. In total, 31 systematic reviews reported on the clinical impact of nonadherence (van Boven et al., 2014; Walsh et al., 2019; Altice et al., 2019; Souza et al., 2016; Lee et al., 2022; Shehab et al., 2019; Sussman et al., 2022; Martin-Ruiz et al., 2018; Evans et al., 2022; Deshpande et al., 2017; Parmar et al., 2017; Capoccia et al., 2016; Ho et al., 2016; De Vera et al., 2014a; Nassetta et al., 2022; Bårnes and Ulrik, 2015; Mikyas et al., 2014; Chimeh et al., 2020; Foka and Mufhandu, 2023; El-Saifi et al., 2018; Ágh et al., 2015; De Vera et al., 2014b; Kengne et al., 2024; Lee et al., 2024; Inotai et al., 2021; Eliassen et al., 2023; Maniadakis et al., 2018; Alahmari et al., 2023; Visintini et al., 2023; Hussain et al., 2021), 12 on the economic impact of nonadherence (Cutler et al., 2018; van Boven et al., 2014; Evans et al., 2022; Deshpande et al., 2017; Capoccia et al., 2016; Ho et al., 2016; Chimeh et al., 2020; Kengne et al., 2024; Maniadakis et al., 2018; Noens et al., 2014; Hameed et al., 2014; Pennington and McCrone, 2018), and 8 on both (van Boven et al., 2014; Evans et al., 2022; Deshpande et al., 2017; Capoccia et al., 2016; Chimeh et al., 2020; Kengne et al., 2024; Maniadakis et al., 2018; Noens et al., 2014) (Figure 1).

Not all studies that were included in the original systematic reviews and meta-analyses focused on both the clinical or economic outcomes. In total, these systematic reviews covered at least 430 unique studies on clinical outcomes and 174 studies on economic outcomes.

The clinical focus of the systematic reviews included mostly patients with cardiovascular disease including atrial fibrillation (*N* = 6) (Shehab et al., 2019; Sussman et al., 2022; Martin-Ruiz et al., 2018; Deshpande et al., 2017; De Vera et al., 2014a; Hameed et al., 2014), hypertension (*N* = 3) (Souza et al., 2016; Kengne et al., 2024; Lee et al., 2024), transplantation (*N* = 4) (Parmar et al., 2017; Nassetta et al., 2022; Visintini et al., 2023; Hussain et al., 2021), and chronic lung diseases (*N* = 3). Importantly, this is not the same as the amount of individual studies in a clinical area. For example, only two systematic reviews on diabetes mellitus were included, but these reviews included about 110 individual studies (Evans et al., 2022; Capoccia et al., 2016), whereas the three reviews on chronic lung disease included maximally 23 individual studies (Bårnes and Ulrik, 2015; Ágh et al., 2015; van Boven et al., 2024). In all studies, the outcomes were compared between nonadherent and adherent patients, or outcomes were compared between different levels of adherence.

Overall, no consistent definition of medication (non)adherence within the included studies was found and in some studies (non)adherence was not defined at all. Also, the adherence measurement methods and thresholds for nonadherence varied greatly within the studies.

### Clinical impact of nonadherence

**FIGURE 2:**
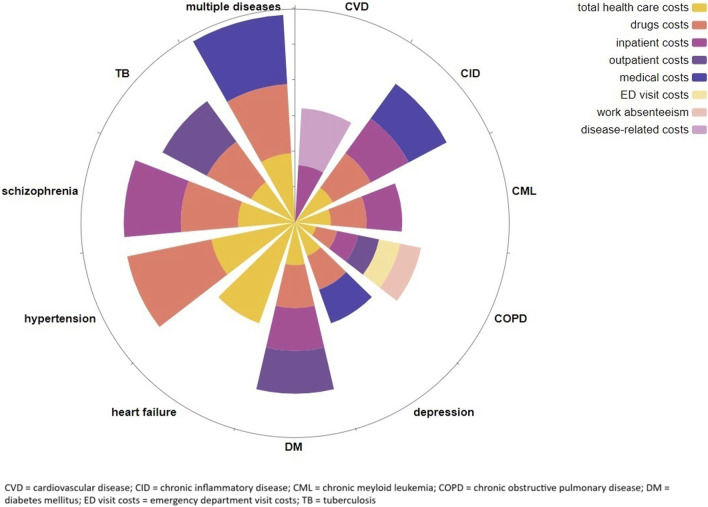
Overview of the clinical consequences of medication nonadherence by clinical area with ray length representing the relative amount of identified studies in each clinical area.

In Table 1, an overview of the systematic reviews on the clinical impact of medication nonadherence is provided. Clinical outcomes were often disease specific, but also more generic outcomes (e.g., hospital admissions, all-cause mortality) were reported to be associated with, and mostly negatively impacted by, medication nonadherence. In Figure 2, the clinical outcomes are summarized by clinical area.

**TABLE 1:** Overview of systematic reviews on the clinical consequences of medication nonadherence.

First author, year	Clinical area	Definition of (non)adherence	Medication	Included studies with clinical outcomes/included studies (n/N)	Clinical outcomes	Direction (-/+)^*^ and signficance S, NS, NR)^**^ on outcome	Effect size^***^
**Walsh et al. (2019)**	Ageing population	“Medication adherence is defined as the process by which patients take their medication as prescribed, consisting of 3 main components: initiation, implementation and discontinuation.”	medication in general, not specified	11/66	hospitalization (all cause)	+ S	OR 1.17 [95% CI, 1.12-1.21) Z=7.65 (p<0.0001)
					hospitalization (disease-specific)	+ NS	OR 1.07 [95% CI, 0.98-1.17)Z=1.47 (p<0.143)
					ED visits	+ NS	OR 1.05 [95% CI, 0.90-1.22] Z=0.57 (p=0.566)
					physician office visits	+ S and NS	
					utilization of outpatient services	+ NS	OR 1.09 (95% CI: 0.87–1.36), (p=0.46)
					quality of life	+ S and NS	
					mortality	+ S and NS	HZ 0.79 95% CI, 0.63-0.98)Z= 2.12 (p=0.034)
					depression	+ NS	
**Shehab et al. (2019)**	Atrial fibribilation (AF)	-	novel oral anticoagulants (NOACs), not specified	6/6	bleeding events	+ S	7.5% (95% CI, 0.2-14.8] (p=0.045)
**Sussman et al. (2022)**	Atrial fibrilation non-vulvar (NVAF) and stroke risk	-	oral anticoagulants, (OACs), not specified	6/16	İschemic events^1^	+ S	
					mortality	+ S and NS	
					bleeding events ^2^	+ S	
**Barnes and Ulrik (2014)**	Asthma	-	ICS (in combination with long-acting β2 agonists), not specified	6/19	number of rescue courses of oral corticosteroids	+ NS	
					hospitalization	+ NS and NR	
					FEV1	- S and NS	
					% eosinophils	- S and NS	
					mortality	+ NR	
**Eliassen et al. (2023)**	Breast cancer	“In breast cancer, treatment non-adherence occurs when a patient fails to take the treatment as prescribed throughout the treatment period (ie, frequently missing doses), whereas non-persistence to AET occurs when a patient stops treatment continuously for a prolonged period of time.”	adjuvant endocrine medication, tamoxifen and aromatase inhibitors	14/14	event-free survival	+ S	
					overall survival	+ S	
**Inotai et al. (2021)**	(nonmetastic) Breast cancer	“... refers to the extent to which a patient acts in accordance with the prescribed interval and dose of a dosing regimen.”	endocrine therapies, not specified	12/12	distant metastasis	+ S	
					recurrence of breast cancer	+ S	
					worse disease free survival	+ S	
					mortality	+ S and NS	
**Deshpande et al. (2017)**	Cardiovascular disease (CVD)	-	statins, not specified	20/139	cardiovascular events ^3^	- S and NS	
					mortality	+ S	
					hospitalization	+ S	
					ED visits	+ S	
**Martin-Ruiz et al. (2018)**	(risk on) Cardiovascular disease (CVD)	-	statins, not specified	17/17	mortality	+ S	
					cardiovascular events ^4^	+ S and NS	
					hospitalization	+ S and NR	
**De Vera et al. (2014a)**	Cardiovascular disease (CVD)	“Medication adherence is a complex construct that encompasses the following distinct problems: (i) poor execution of the dosing regimen, such that doses are delayed or omitted, which may lead to transient interruptions in drug action; and (ii) discontinuation of the medication, which may lead to intermittent or permanent loss of drug effects.”	statins, not specified	19/28	cardiovascular events ^5^	+ S	
					mortality	+ S	
					hospitalization	+ S	
**Maniadakis et al. (2018)**	Chronic inflammatory disease (CID)	-	biologic therapy (TNF)	7/17	disease activity	+ S and NR	
					disease relapse	+ NR	
					disease duration	+ NR	
					hospitalization	+ NR	
**Noens et al. (2014)**	Chronic myeloid leukemia (CML)	-	BCR-ABL inhibitor therapy, imatinib	6/19	suboptimal response	+ S	
					event-free survival	- S	
**Ágh et al. (2015)**	Chronic obstructive pulmonary disease (COPD)	“Medication adherence ‘refers to the act of conforming to the recommendations made by the provider with respect of timing, dosage and frequency of medication taking’.”	COPD medication, not specified	7/7	quality of life	- S and NS	
**Van Boven et al. (2014)**	Chronic obstructive pulmonary disease (COPD)	“… the extent to which a patient acts in accordance with the prescribed interval and dose of a dosing regimen”	COPD medication, not specified	7/12	hospitalization	+ S, NS and NR	
					ED visits	+ NS and NR	
					outpatient visits	+ NS and NR	
					symptoms ^6^	+ NR	
					FEV1	- NS	
					PC20	- S	
					mortality	+ S and NS	
					quality of life	- S and NS	
**Ho et al. (2016)**	Depressive disorder	-	antidepressants, not specified	9/11	relapse or recurrence	+ S	
					ED visits	+ S and NR	
					hospitalization	+ S	
					depression severity	+ S	
					response and remission	- S	
**El-Saifi et al. (2018)**	Dementia	-	not specified	1/20	institutionalisation	+ NR	
					mortality	+ NR	
**Capoccia et al. (2016)**	Diabetes mellitus (DM)	-	glucose-lowering agents, not specified	12/98	HbA1c	- S	
					diabetic complications	+ S	
					ED visits	+ S	
					hospitalization	+ S	
**Evans et al. (2022)**	Diabetes mellitus (DM) type 2	-	antidiabetic medications, not specified	81/92	HbA1c	- S and NS	
					hypoglycaemia	- S and NS	
					hospitalization	+ - NR	
					ED visits	+ - NR	
					outpatient visits	+ - NS or NR	
					microvascular events ^7^	+ S and NS	
					macrovascular events ^8^	+ S and NS	
**Vera, 2014**	Gout	-	allopurinol and uric acid lowering agents, not specified	1/16	sUA concentration	- NR	
**Visintini et al. (2023)**	Haematopoietic stem cell transplantation (HSCT)	“…the late or non-initiation of the prescribed treatment, sub-optimal implementation of the dosing regimen, or early discontinuation of the treatment.”	immunosuppressents, not specified	5/14	GvHD	+ NS	
					mortality	+ NS	
**Hussain et al. (2021)**	Heart transplantation (HTx)	**-**	immunosuppresents, not specified	3/23	transplant coronary artery disease	+ S	
					acute late rejection	+ NS	
					mortality	+ S and NS	
**Nassetta et al. (2022)**	(pediatric) Heart transplantation (HTx)	-	İmmunosuppressents, not specified	11/14	transplant rejection	+ S and NS	
					hospitalization	+ S	
					mortality	+ S	
					quality of life	- S	
					mental health	- S and NS	
**Altice et al. (2019)**	Human immunodeficiency virus (HIV)	-	antiretroviral therapies (ARTs), not specified	18/29	viral suppresion	- S and NS	
**Foka and Mufhandu (2023)**	Human immunodeficiency virus (HIV)	-	antiretroviral therapies (ARTs), not specified	NR/176	virologic failure	+ NR	
**Souza et al. (2016)**	Hypertension, arterial	-	antihypertensive medication, not specified	4/20	quality of life	- S	MD 9.24 [95% CI, 8.16-10.33], Z=16.71 (p<0.00001)
**Lee et al. (2022)**	Hypertension	-	antihypertensive medication, not specified	53/162	systolic BP control	- S	MD 3.76 mm Hg [95% CI, 2.23–5.28 mm Hg] (p<0.001)
					diastolic BP control	- S	MD 3.11 mm Hg [95% CI, 2.24–3.99 mm Hg] (p<0.001)
					BP control	- S	OR 2.15 [95% CI, 1.84–2.5] (p<0.001)
					complications from hypertension	+ S	OR 2.08 [95% CI, 0.99–4.35] (p<0.001)
					hospitalization	+ NS	OR 1.38 [95% CI, 1.35–1.41] (p=0.64)
					mortality	+ NS	OR 1.38 [95% CI, 1.35–1.41] (p=0.509)
**Kengne et al. (2024)**	Hypertension and/or dyslipidemia	-	antihypertensives and lipid-lowering medications, not specified	45/45	BP control LDL	- S - S	
					cardiovascular events ^9^	+ S and NS	
					mortality	+ S and NR	
**Parmar et al. (2017)**	(pediatric) Liver transplantation (LTx)	-	tacrolimus	3/25	quality of life	- S	
**Alahmari et al. (2023)**	Osteoporosis	“Adherence is sometimes used interchangeably with compliance or as a more general term to refer to both compliance and persistence.”	osteoporotic medication, not specified	14/14	fracture risk	+ NR	
					bone mineral density	- NR	
**Mikyas et al. (2014)**	(male) Osteoporosis	-	bisphosphonates, not specified	3/18	fracture risk	+ NR	
**Lee et al. (2024)**	Thalassaemia	-	İron-chelation agents, not specified	20/20	serum ferritin	- S and NS	
					liver disease	+ S	
					liver iron overload	+ S and NS	
					cardiac disease	+ S	
					cardiac iron overload	+ S and NS	
					endocrinologic morbidity	+ S	
					hepatic morbidity	+ S	
					quality of life	- S sand NS	
**Chimeh et al. (2020)**	(drug-susceptible) Tuberculosis (TB)	“Adherence is defined as “the extent to which a person’s behavior to take medicines, to follow a diet, and/or to execute lifestyle changes corresponds with agreed recommendations from a healthcare provider.”	TB medication, not specified	12/14	unsuccessful treatment	+ S and NS	
					successful treatment	- NS and NR	
					mortality	+ S and NS	

^*^+ = positive relation between clinical outcome and nonadherence; - = negative relation between clinical outcome and nonadherence.

^**^S = significant, NS, nonsignificant; NR, not reported.

^***^ in z-score (p-value), mean-difference (p-value) or odds ratio (p-value).

^1^ ischemic events = central and non-central nervous system embolism, ischemic strokes, TIA, tromboembolism

^2^ bleeding events = hemorraghic stroke, major bleeding, gastrointestinal hemorrhaging.

^3^ cardiovascular evenets = IHD, non-fatal CAD, AMI.

^4^ cardiovascular events = i.e., AMI, ACS, CAD, CeVD, CHD, CHF, CVD, HF, IHD, MI, stroke.

^5^ cardiovascular events = ACS, AMI, CVD, CAD, CHF, CeVD, VTE.

^6^ symptoms = cough, phlegm, dyspnea.

^7^ microvascular events = amputations/ulcers nephropathy, neuropathy, renal failure, retinopathy, PVD.

^8^ macrovascular events = angina, angioplasty, CABG, CeVD, CeV complications, CVD, CV, complications, CHF, HF, IHD, MI, stroke, TIA.

^9^ cardiovascular events = CAD, overall CVD, acute CVD, CeVD, HF, CHF, IHD, AMI, stroke TIA.

Abbreviations: ACS, acute coronary syndrome; AMI, acute myocardial infarction; ART, antiretroviral therapy; BP, blood pressure; CABG, coronary artery bypass graft; CAD, coronary artery disease; CeV complications, cerebrovascular complications; CeVD, cerebrovascular disease; CHD, chronic heart disease; CHF, chronic heart failure; CV, complications, cardiovascular complications; ED, visits, emergency department visits; GvHD, graft versus host disease; HF, heart failure; HSCT, haematopoietic stem cell transplantation; ICS, inhaled corticosteroids; IHD, ischemic heart disease; MI, myocardial infarction; sUA, serum uric acid; TIA, transient ischemic attack; PVD, peripheral vascular disease; VTE, venous thromboembolism.

### Generic clinical outcomes

The generic clinical outcomes (mostly) significantly impacted by nonadherence that were identified within the systematic reviews were quality of life (*N* = 7) (van Boven et al., 2014; Souza et al., 2016; Parmar et al., 2017; Nassetta et al., 2022; Bårnes and Ulrik, 2015; Chimeh et al., 2020; Ágh et al., 2015), hospitalization (*N* = 12) (van Boven et al., 2014; Walsh et al., 2019; Lee et al., 2022; Martin-Ruiz et al., 2018; Evans et al., 2022; Deshpande et al., 2017; Capoccia et al., 2016; Ho et al., 2016; De Vera et al., 2014a; Nassetta et al., 2022; Bårnes and Ulrik, 2015; Maniadakis et al., 2018), emergency department (ED) visits (*N* = 5) (van Boven et al., 2014; Walsh et al., 2019; Evans et al., 2022; Capoccia et al., 2016; Ho et al., 2016), outpatient visits (*N* = 2) (van Boven et al., 2014; Evans et al., 2022)and mortality (*N* = 15) (van Boven et al., 2014; Walsh et al., 2019; Lee et al., 2022; Sussman et al., 2022; Martin-Ruiz et al., 2018; Deshpande et al., 2017; De Vera et al., 2014a; Nassetta et al., 2022; Bårnes and Ulrik, 2015; Chimeh et al., 2020; El-Saifi et al., 2018; Kengne et al., 2024; Inotai et al., 2021; Visintini et al., 2023; Hussain et al., 2021).

In patients with arterial hypertension (Souza et al., 2016), asthma (Bårnes and Ulrik, 2015), COPD (van Boven et al., 2014; Ágh et al., 2015), thalassemia (Lee et al., 2024), and in (pediatric) patients with a heart or liver transplantation (Parmar et al., 2017; Nassetta et al., 2022), quality of life was overall negatively associated with medication nonadherence. However, whether a lower quality of life resulted in more medication nonadherence or *vice versa* was not always clear. Besides, although all the reviews focused on health related quality of life (HRQoL) it was measured variously over the included studies and in some studies and reviews a distinction was made between different components of quality of life. For example, in the review concerning patients with hypertension, both the total scores on quality of life as well as the mental and physical component were presented (Souza et al., 2016). In table 1, this differentiation has not been made and only the overall impact on quality of life is presented.

A higher risk of hospitalization due to nonadherence was found in more patient populations, that is, in patients with asthma (Bårnes and Ulrik, 2015), chronic inflammatory disease (Maniadakis et al., 2018), COPD (van Boven et al., 2014), cardiovascular disease (Martin-Ruiz et al., 2018; Deshpande et al., 2017; De Vera et al., 2014a), depression (Ho et al., 2016), diabetes (Capoccia et al., 2016), pediatric heart transplantation (Nassetta et al., 2022), hypertension (Lee et al., 2022), and in a general aging population (Walsh et al., 2019). Hospitalization was also operationalized variously, i.e., hospital admissions, duration of being hospitalized, or specified as being disease-specific or all-cause hospitalization. The latter differentiation was also found in relation to the outcomes “outpatient visits” and Emergency Department (ED) visits. The systematic reviews that covered these clinical outcomes showed an increase in ED visits (van Boven et al., 2014; Walsh et al., 2019; Evans et al., 2022; Capoccia et al., 2016; Ho et al., 2016) and outpatient visits (van Boven et al., 2014; Walsh et al., 2019; Evans et al., 2022) in nonadherent patients with COPD (van Boven et al., 2014), diabetes (Evans et al., 2022; Capoccia et al., 2016), depression (Ho et al., 2016) and in the general aging population (Walsh et al., 2019). Notably, one systematic review on patients with type 2 diabetes demonstrated less outpatient visits in patients being less nonadherent to their medication compared to patients that were more adherent (Evans et al., 2022). One systematic review found a higher risk on institutionalization in nonadherent patients with dementia (El-Saifi et al., 2018).

Furthermore, it was found that mortality rates, all-cause or disease specific, were higher in nonadherent patients with non-vulvar atrial fibrillation (Sussman et al., 2022), asthma (Bårnes and Ulrik, 2015), breast cancer (Inotai et al., 2021; Eliassen et al., 2023), COPD (van Boven et al., 2014), cardiovascular disease (Martin-Ruiz et al., 2018; Deshpande et al., 2017; De Vera et al., 2014a), dementia (El-Saifi et al., 2018), heart and stem cell transplants (Nassetta et al., 2022; Visintini et al., 2023; Hussain et al., 2021), hypertension (Lee et al., 2022; Kengne et al., 2024) and tuberculosis (Chimeh et al., 2020). In patients with breast cancer, it was also found that the probability of disease-free survival was higher among more adherent patients (Inotai et al., 2021).

### Disease specific clinical outcomes

Some studies included disease specific outcomes such as disease-specific health risks or complications, disease-related symptoms or disease-specific biomarkers.

In patients with COPD (van Boven et al., 2014), chronic inflammatory diseases (CID) (Maniadakis et al., 2018) and depression (Ho et al., 2016) and the aging population (Walsh et al., 2019), disease-specific symptoms, such as experienced depression severity, were used as a clinical outcome. Overall, these patients reported either more symptoms or more severe symptoms when they were not adhering to their medication regimen. One review reported an association between patient medication behavior as clinical outcome and nonadherence (Bårnes and Ulrik, 2015). That is, in patients with asthma, a higher number of rescue courses of oral corticosteroids (a proxy for asthma exacerbations) was positively associated with nonadherence, although nonsignificant (Bårnes and Ulrik, 2015).

Disease-related health risks and complications were mostly reported in patients with cardiovascular disease. Generally, these patients had a significant higher risk for cardiovascular events such as acute myocardial infarction (AMI), cerebrovascular disease (CeVD), and ischemic stroke, when being nonadherent compared to patients being adherent (Martin-Ruiz et al., 2018; Deshpande et al., 2017; De Vera et al., 2014a; Kengne et al., 2024). On the contrary, in patients with AF being adherent to anticoagulants, this was significantly and positively associated with bleeding events (Shehab et al., 2019; Sussman et al., 2022). In patients with heart, liver or stem cell transplantation (Nassetta et al., 2022; Visintini et al., 2023; Hussain et al., 2021), patients with breast cancer (Inotai et al., 2021; Eliassen et al., 2023), HIV (Foka and Mufhandu, 2023), hypertension (Lee et al., 2022; Kengne et al., 2024), thalassemia (Lee et al., 2024), tuberculosis (Chimeh et al., 2020) and DM (Evans et al., 2022; Capoccia et al., 2016), being nonadherent was mostly significantly related to severe and life-threatening disease-specific complications as well. Furthermore, recurrence or worsening of disease was more common in nonadherent patients with breast cancer (Inotai et al., 2021), CID (Maniadakis et al., 2018) and depression (Ho et al., 2016). Lastly, it was demonstrated that patients with osteoporosis have a significantly higher risk of fractures (Mikyas et al., 2014; Alahmari et al., 2023) and patients with hypertension have significantly worse blood pressure control (Lee et al., 2022; Kengne et al., 2024) due to medication nonadherence.

Biomarkers have been associated with nonadherence such as bone mineral density in osteoporosis (Alahmari et al., 2023) or forced expiratory volume in 1 s (FEV1) (van Boven et al., 2014), eosinophil percentage (Bårnes and Ulrik, 2015) in asthma and additionally histamine determination (PC20) (van Boven et al., 2014) in COPD. Other biomarkers that have been negatively influenced by nonadherence are glycohemoglobin (HbA1c) (Evans et al., 2022; Capoccia et al., 2016) and hypoglycaemia (Evans et al., 2022) in DM, serum urine acid (sUA) in gout (De Vera et al., 2014b; Lee et al., 2024), serum ferritin in thalassaemia (Lee et al., 2024), and blood pressure control (Lee et al., 2022; Kengne et al., 2024) and cholesterol levels (LDL) (Kengne et al., 2024) in hypertension.

### Economic impact of nonadherence

**FIGURE 3:**
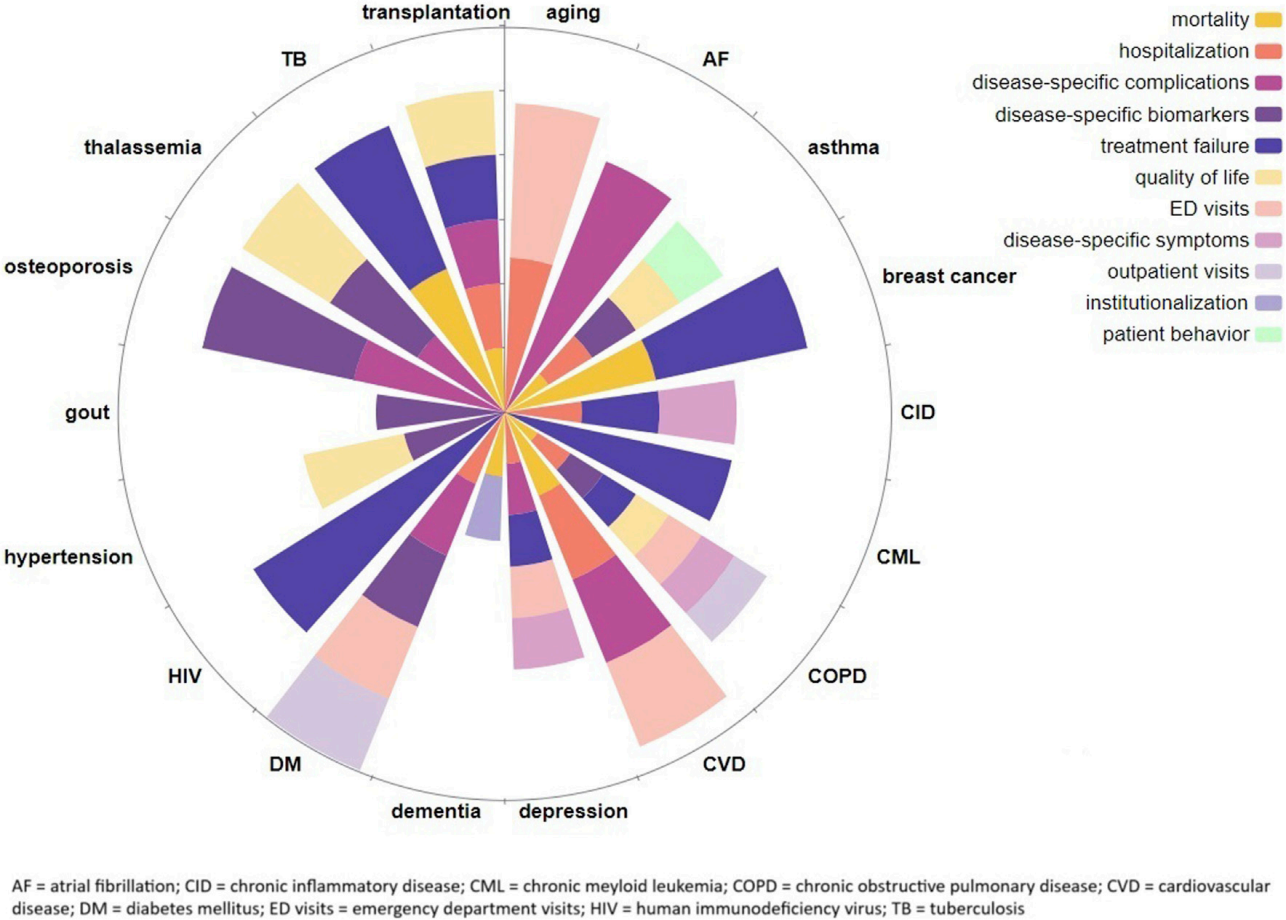
Overview of the economic consequences of medication nonadherence by clinical area with ray length representing the relative amount of identified studies in each clinical area.

In Table 2, an overview of the economic impact of medication nonadherence is provided. Economic outcomes were often direct healthcare costs, though only one systematic review provided data on the impact of medication nonadherence on indirect costs. In Figure 3, the economic outcomes are summarized by clinical area.

**TABLE 2:** Overview of systematic reviews reporting on the economic consequences of medication nonadherence.

First author, year of publication	Clinical area	Definition of (non)adherence	Medication	Included studies with economic outcomes/included studies (n/N)	Economic outcomes	Direction (-/+)* and significance (S, NS, NR) ** on outcome
Deshpande (2017)	Cardiovascular disease (CVD)	“Adherence is usually defined as the extent to which a patient acts in accordance with the prescribed interval and dosing regimen.”“Persistence is defined as the duration of time from the initiation to discontinuation of therapy.”	statins, not specified	3/151	inpatient costs	- NR
					other CVD related costs	- NR
Maniadakis (2018)	Chronic inflammatory disease (CID)	“Compliance: The extent to which a patientacts in accordance with the prescribed interval and dose of a dosing regimen.”“Persistence: The duration of time from initiation to discontinuation of therapy.”	biologic therapy	7/129	drug costs	+ NR
					inpatient costs	- S
					medical costs	- NR
					total healthcare costs	- S
Noens (2014)	Chronic myeloid leukaemia (CML)	-	BCR-ABL inhibitor (imatinib)	3/19	drug costs	+ NR
					inpatient costs	- NR
					total healthcare costs	- NR
van Boven (2014)	Chronic obstructive pulmonary disease (COPD)	“the extent to which a patient acts in accordance with the prescribed interval and dose of a dosing regimen”	COPD medication, not specified	4/12	drug costs	+ NR
					inpatient costs	- NR
					outpatient costs	+ - NR
					ED visits costs	- NR
					total healthcare cost	- NR
					work absenteeism	- S
Ho (2016)	Depressive disorder	-	antidepressants, not specified	3/11	drug costs	+ S
					medical costs (physician, emergency room, hospital, laboratory, or any other medical charges)	- S and NS
					total healthcare costs	+ - NS
Capoccia (2015)	Diabetes mellitus (DM)	-	glucose-lowering agents, not specified	4/98	inpatient costs	- S
					total healthcare costs	- S and NR
Evans (2021)	Diabetes mellitus (DM), type 2	“adherence as the extent to which a person's antidiabetic medication-taking behaviour corresponds with recommendations from their healthcare provider”“Persistence was estimated based on the fill time between prescriptions or medication insurance claims.”	antidiabetic medications, not specified	20/92	drug costs	+ S and NS, - NS
					inpatient costs	- S and NS
					outpatient costs	- S and NS
					other costs	+ - NS
					total healthcare costs	+ - S and NS
Hameed (2014)	Heart failure	“the extent to which a person’s behaviour - taking medication, following a diet, and/or executing lifestyle changes, corresponds with agreed recommendations from a health care provider”	not specified	3/9	total healthcare costs	- NR and NS
Kengne (2024)	Hypertension and/or dyslipidaemia	-	antihypertensives and lipid-lowering medications, not specified	18/45	drug costs	+ NR
					total healthcare costs	- S
Pennington (2018)	Schizophrenia	-	antipsychotics, not specified	28/28	drug costs	- S and NS, + S
					inpatient costs	+ - S and NS
					total healthcare costs	+ - S and NS
Chimeh (2020)	(drug-susceptible) Tuberculosis (TB)	“Adherence is defined as “the extent to which a person’s behaviour to take medicines, to follow a diet, and/or to execute lifestyle changes corresponds with agreed recommendations from a healthcare provider.”	TB medications, not specified	2/14	drug costs	+ NR
					outpatient costs	+ NR
					total healthcare costs	+ - NR
Cutler (2018)	Multiple diseases	“the extent to which the patients’ behaviour matches agreed recommendations from the prescriber”	not specified	79/79	drug costs	+ NR
					medical costs	+ - NR
					total healthcare costs	+ - NR

*+ = positive relation between clinical outcome and nonadherence; - = negative relation between clinical outcome and nonadherence, ? = direction of relation between clinical outcome and nonadherence not stated or unclear

** S= significant, NS= nonsignificant, NR= not reported

Abbreviations: CID=chronic inflammatory disease; CML=chronic myeloid leukaemia; COPD=chronic obstructive pulmonary disease; CVD= cardiovascular disease; DM= diabetes mellitus; ED visit= emergency department visit

#### Direct healthcare costs

Direct healthcare cost outcomes identified within the included systematic reviews were inpatient costs (*N* = 7), outpatient costs (*N* = 3) (van Boven et al., 2014; Evans et al., 2022; Chimeh et al., 2020), ED visit costs (*N* = 1) (van Boven et al., 2014), medical costs (healthcare costs excluding drug costs) (*N* = 2) (Ho et al., 2016; Maniadakis et al., 2018), drug costs (*N* = 8) (van Boven et al., 2014; Evans et al., 2022; Ho et al., 2016; Chimeh et al., 2020; Kengne et al., 2024; Maniadakis et al., 2018; Noens et al., 2014; Pennington and McCrone, 2018), and total healthcare costs (*N* = 11) (Cutler et al., 2018; van Boven et al., 2014; Evans et al., 2022; Capoccia et al., 2016; Ho et al., 2016; Chimeh et al., 2020; Kengne et al., 2024; Maniadakis et al., 2018; Noens et al., 2014; Hameed et al., 2014; Pennington and McCrone, 2018).

In most reviews, nonadherent patients had higher medical costs and lower drug costs. However, the overall impact of medication nonadherence on total healthcare costs was found to be mixed, mostly varying between increased costs and no significant change. This variation depended on whether the higher medical costs were balanced by the lower spending on drugs. Similar trends could be observed across all investigated disease areas, including DM (Evans et al., 2022; Capoccia et al., 2016), tuberculosis (Chimeh et al., 2020), cardiovascular disease (Deshpande et al., 2017), heart failure (Hameed et al., 2014), hypertension and dyslipidaemia (Kengne et al., 2024), depressive disorder (Ho et al., 2016), schizophrenia (Pennington and McCrone, 2018), chronic inflammatory disease (Maniadakis et al., 2018), chronic myeloid leukaemia (Noens et al., 2014), and COPD (van Boven et al., 2014).

The economic impact of medication nonadherence across multiple disease groups was evaluated in one systematic review (Cutler et al., 2018) only. This review revealed that medication nonadherence was generally associated with higher total healthcare costs, with significant variability in the economic impact across different diseases. Specific estimates for the mean (SD) adjusted total cost of medication nonadherence per annum per person were as follows: DM at $8,327 ($2,335), respiratory disease at $8,584 ($469), cardiovascular disease at $12,146 ($5,320), mental health conditions at $14,585 ($5,315), gastrointestinal disease at $30,771 ($8,270), and osteoporosis at $43,372 ($14,266) (all costs adjusted to 2024 US$). Despite the fact that cost data across various disorders were compared after being converted to the same currency and year, and were extrapolated to annual costs, there was a wide range between disease-specific estimates. This variability can be partly attributed to the various cost indicators used by the individual studies and other heterogeneity in study design, not allowing meaningful cost comparisons between diseases.

#### Indirect costs

Only one systematic review (van Boven et al., 2014) reported data on the association between medication adherence and indirect costs, specifically productivity. Based on a retrospective analysis of US administrative healthcare claims, adherent patients with asthma/COPD had significantly fewer days absent from work, with potential annual savings of around $2,504 (adjusted to 2024 US$) per employee. Cutler et al. (2018) also investigated indirect costs, but did not report any information on the impact of medication nonadherence on indirect costs, only which study assessed them and which types of indirect cost outcomes were included (e.g., short-term disability, workers’ compensation, paid time off costs, productivity costs, absenteeism costs, and presenteeism costs).

## Discussion

### Main findings

This narrative review of systematic reviews demonstrates the many negative, and sometimes even fatal, consequences of medication nonadherence. Thirty-one systematic reviews on the association between nonadherence and clinical outcomes were found across 17 different clinical areas and 12 systematic reviews on the association with economic outcomes in 11 clinical areas. Most studies on clinical outcomes demonstrated a positive and significant association between nonadherence and mortality, hospitalization and ED visits. Areas covered were mostly organ transplantation, cardiovascular diseases, and chronic lung diseases. Almost all studies on economic outcomes showed higher costs in patients with lower levels of adherence to medication. These costs were mostly related to total healthcare costs, drug costs and inpatient costs.

### Interpretation of findings

Nonadherence demonstrated to negatively impact most identified outcomes significantly over multiple studies and clinical areas, and with respect to both economic and clinical outcomes. Given this broad impact, there are some important considerations that need to be highlighted.

Medication nonadherence was regularly not (clearly) defined and measurement methods varied greatly within and between outcomes and clinical areas. This is a well-known described issue in both research and daily clinical practice (Jimmy and Jose, 2011; Lam and Fresco, 2015; Stirratt et al., 2015). Besides, each measurement method is known for its unique strengths and limitations, e.g., the questionable reliability of patient self-reports and the limited informational value but more objectiveness of pharmacy records (Jimmy and Jose, 2011; Lam and Fresco, 2015). Because of these limitations, it has been recommended to combine two different measurement methods for optimal and reliable information on patients’ medication use (Lam and Fresco, 2015). To obtain more detailed and objective data, digital adherence technology such as electronic pill bottles or digital inhalers could be used. Yet, most studies used only one measurement method. Remarkably, one study used clinical outcomes itself (i.e., reduction or control of blood pressure) as (indirect) measurement method of level of adherence (Souza et al., 2016). Furthermore, two studies differentiated between medication adherence and persistence (Evans et al., 2022; Maniadakis et al., 2018). Even though persistence always includes the element of time in its measurement method and adherence measurement methods do not, we did not use this differentiation in our review. When we referred to adherence in this review, this also included persistence. Altogether, the variety in measurement methods of medication adherence is both a strength and a limitation. On the one hand, even when measured differently, similar findings are demonstrated across studies, confirming and strengthening the evidence regarding the negative impact of nonadherence. On the other hand, due to this variety in measurement methods, comparisons within the same clinical area but across different studies, is challenging and meta-analysis was often not possible. Note that also outcome definitions varied, i.e., hospitalization was measured as rehospitalization, duration of hospitalization, all-cause hospitalization and disease-related hospitalization.

With this review, we aimed to provide an overview of the consequences of medication nonadherence. However, it was not always possible to clearly distinguish between the consequences and the associated factors of medication adherence. Causality is difficult to establish, even in clinical trials. Where most criteria for demonstrating a causal relation are integrated in randomized controlled trials, it is not bulletproof, especially when it concerns patient behavior or experiences. An example of a clinical outcome for which causality with adherence is questionable, is quality of life. If medication adherence improves quality of life–due to, e.g., less symptoms as consequence of medication adherence–or if a higher quality of life results in more medication adherent behavior–because patients with a higher quality of life, for example, receive more social support to be adherent.

Related to the topic of causality, an absence of treatment effect–seen in, e.g., patients with HIV, tuberculosis, COPD and asthma–and negative consequences of pharmaceutical treatment–e.g., in patients with AF–can also be caused by individual differences in pharmacokinetics and pharmacodynamics. For example, some studies identified a lower probability of virologic suppression in nonadherent HIV patients compared to adherent HIV patients (Altice et al., 2019; Foka and Mufhandu, 2023). In another study, no significant difference in improvement in lung function–FEV1 in COPD and asthma, and % eosinophils in asthma–was found between adherent and nonadherent COPD and asthma patients (van Boven et al., 2014; Bårnes and Ulrik, 2015). The treatment failure in these studies could indeed be attributed to medication nonadherence, however treatment failure could potentially also be (partially) explained by the absence of a biological response in these patients. Regarding these individual differences in biological response, in some clinical areas relatively little is known yet, e.g., asthma and depression (Drevets et al., 2022; Vijverberg et al., 2018; Norbury and Seymour, 2018), and this variability is not accounted for in medication effectiveness and adherence studies. However, an increase in interest is seen in, e.g., studies on the biological response differences between men and women (Soldin and Mattison, 2009; Madla et al., 2021; Franconi et al., 2007).

The relevance of medication adherence differs greatly between diseases and there seems to be relatively limited attention for these characteristics. The forgiveness of a drug concerns the amount of deviation in adherence that is allowed to still gain the intended effect of that drug (Assawasuwannakit et al., 2015). This pharmaceutical forgiveness of nonadherence, and the threshold of medications’ treatment effect varies by disease and drug (McAllister et al., 2022; Osterberg et al., 2010). For example, the forgiveness of nonadherence for immunosuppressants in patients with organ transplantation is much lower than the forgiveness of statins in patients with (a risk for) cardiovascular diseases (Osterberg et al., 2010). Although we identified mostly severe to fatal consequences of nonadherence, also the clinical and economic consequences of medication nonadherence can be more or less severe, and therefore more or less relevant, across clinical areas. The relevance–forgiveness and consequences–of medication nonadherence for a disease is an essential consideration when comparing medication nonadherence over multiple clinical areas (McAllister et al., 2022). The same is true for the feasibility to achieve good medication adherence. Medication plans or schedules can be more or less complex and extensive within and between both individual patients and diseases. The higher the impact and likelihood of nonadherence in any particular disease area, the higher the likelihood that interventions that focus on enhancing adherence will be clinically effective and cost-effective.

### Strengths and limitations

With this semi-systematic narrative review, we have aimed to provide an up-to-date overview of the overall impact of medication nonadherence across disease areas. However, given the pragmatic nature of this narrative review, we possibly missed some relevant articles with our search strategy as we only included articles published in English, and focused on systematic reviews published in the last decade. Also, some studies could have been overlooked given only one researcher included the studies on clinical outcomes and one researcher included the studies on economic outcomes. Also, we do not provide a detailed overview of the included studies. These details, such as the medication adherence measurement methods, are important for the interpretation of our findings. However, we do provide an extensive overview of all the clinical outcomes together with how these outcomes are associated with nonadherence and its significance. In addition, the context–such as specific patient characteristics (e.g., health literacy) or the organization of healthcare in a specific population (e.g., accessibility) – could potentially moderate the relation between nonadherence and the clinical and economic consequences. This was however beyond the scope of this review. Notably, we also categorized negative consequences as reported in the included reviews. However, whether the consequence is indeed always negative or positive depends on its context. For example, in one study on patients with diabetes, it was found that nonadherence was related to less outpatients visits. Whether less frequent outpatient visits are however negative for the patient depends on the nature of these visits. That is, if the outpatient visits concern pro-active or preventative disease and medication patient behaviors, more outpatient visits could not be interpreted as negative. This should be considered when interpreting the these study’s findings. Furthermore, although we only included systematic reviews and meta-analysis published in the last decade, these studies mostly included original studies that were published before. Another strength is that we included different clinical specialties instead of focusing on one clinical area as in most previous reviews. This allows some comparison across diseases, and could contribute to identify priority clinical areas in which nonadherence should be addressed and tackled.

### Recommendations

The clinical consequences for patients and the financial burden of medication nonadherence has been established once more, demonstrating the necessity to invest in interventions detecting and managing nonadherence. However, it remains a challenge for healthcare providers to identify and manage treatment nonadherence (Jimmy and Jose, 2011; El Halabi et al., 2022). More use of (a combination of) validated and objective adherence measurement instruments and the implementation of effective interventions in policy and in daily clinical practice is recommended (Jimmy and Jose, 2011; Lam and Fresco, 2015). However, implementation often turns out to be challenging given studies typically report limited details necessary for implementation (Zullig et al., 2019). We recommend contacting study teams of relevant literature on effective interventions to provide this necessary information. In reporting, implementation science and frameworks can be used to determine what information is needed (Bauer and Kirchner, 2020). Most importantly, the interventions should consider the implementation process from the start, i.e., including stakeholders in the whole process and report more details on the context. Furthermore, concordance of patients’ and physicians’ treatment goals) and simplification of treatment regimens–where possible–are highly recommended for managing nonadherence (El Halabi et al., 2022). Yet most importantly, the reasons for nonadherence should be used as guide for selecting the most suitable intervention. The communication skills of healthcare professionals are demonstrated to be crucial in this and are often demanded to execute interventions effectively (Haskard Zolnierek and DiMatteo, 2009). Educational programs and intervention trainings should therefore emphasize verbal and nonverbal communication skills. In this review, several inconsistencies and gaps in the literature were identified and this provides guidance for further research. Primarily, there is a need for more disease-specific differentiation between adherence and nonadherence and its measurement methods. Future studies on the impact of medication nonadherence should consider the pharmaceutical forgiveness of each specific pharmaceutical treatment–including the threshold for the treatment effect–to create a more meaningful differentiation between adherence and nonadherence. This together with a unified definition of medication nonadherence will also allow for a more meaningful comparison between studies and clinical areas, and could provide essential insights to inform treatment guidelines. Besides, a more in depth understanding of some disease-specific causes and its influences on nonadherence is required, e.g., on the possible influence of heterogeneity in biological response on medication nonadherence and its clinical consequences. Lastly, although many interventions for nonadherence have been developed over the years, there is still a need for more precise and usable adherence measurements that can be integrated into daily clinical practice (Jimmy and Jose, 2011; Lam and Fresco, 2015). The more specific and valid medication nonadherence measures, the more relevant these measures are for daily practice and therefore the higher the change of uptake of these measure in guidelines and practice. Evidently, though some of the chronic diseases with the highest disease burden are covered–i.e., cardiovascular disease, diabetes mellitus and chronic lung diseases COPD and asthma–there are also clinical areas that were less covered. Although a worldwide increase is found in, e.g., mental health diseases, cancers and substance use, relatively little or no studies concerning these clinical areas were identified and covered in this narrative review (United Nations, 2023; Roser et al., 2024). More research in needed in these clinical areas. Moreover, contextual factors such as population specific characteristics (e.g., health beliefs) or healthcare organizational factors (e.g., accessibility of healthcare) could potentially also moderate the relationship between nonadherences and the consequences differently in various disease groups. This was beyond the scope of this study and seems to be an underexposed although potentially relevant topic for further comparison between disease groups.

Despite the amount of studies demonstrating the serious consequences of medication nonadherence, the rates of nonadherence do not seem to have declined although it is estimated that medication use and costs will keep increasing the next years (IQVIA Institute, 2024). A positive remark is that adherence issues have been integrated more and more in guidelines, e.g., in the GINA 2023 report on asthma and the ESC 2024 guidelines on hypertension (McEvoy et al., 2024; Global Initiative for Asthma, 2023). Still, we should bring nonadherence to the top of the agenda of stakeholders. We should focus particularly on the implementation of nonadherence measurement instruments and interventions thereby taking into account the socioeconomic and cultural factors associated with nonadherence. The socioeconomic and cultural factors such as lack of access to medicines due to lack of financial capacity or reachability of healthcare facilities, but also the reluctance of patients to embrace medication regimes because of, e.g., cultural differences, are crucial (Ágh et al., 2024). In many African countries and in Traditional Chinese Medicine, spirituality and herbal products have a more prominent healthcare and the patients’ health beliefs. Therefore, the negative consequences of medication nonadherence observed in this review could be worse in developing regions and regions with different beliefs and customs concerning healthcare such as Sub-Saharan Africa (Kagee et al., 2011; Macquart de Terline et al., 2019). Lastly, both in designing and implementing nonadherence interventions, the context should be considered thoroughly and measurement instruments and interventions should be adjusted culturally appropriate.

## Conclusion

Across disease areas, medication nonadherence in patients with chronic diseases has been associated with elevated disease burden and mortality, increased healthcare utilization (including hospital admissions), and higher direct (e.g., more healthcare provider visits) and indirect financial cost burden (e.g., work productivity losses due to absenteeism and presenteeism). Given this significant impact, interventions on both policy, health system and individual patient level are required. For the greater implementation of measurement instruments and interventions in daily practice, stakeholders such as healthcare professionals, patients and insurers need to be involved from the start. Current available evidence to improve nonadherence could be used more effectively by considering the context and content of both the studies and the targeted population more thoroughly. Furthermore, the development and more frequent and precise use of adherence measurement tools, the provision of personalized interventions based on nonadherence behavioral phenotypes and adequate reimbursement of cost-effective adherence enhancing interventions in daily practice are recommended.
