# Correction: Clinical and economic consequences of medication nonadherence: a review of systematic reviews

**DOI:** 10.3389/fphar.2026.1822926

**Published:** 2026-03-12

**Authors:** Maria Achterbosch, Nilay Aksoy, George D. Obeng, David Ameyaw, Tamas Ágh, Job F. M. van Boven

**Affiliations:** 1 Department of Clinical Pharmacy and Pharmacology, University Medical Center Groningen, University of Groningen, Groningen, Netherlands; 2 Department of Clinical Pharmacy, School of Pharmacy, Altinbas University, Istanbul, Türkiye; 3 Syreon Research Institute, Budapest, Hungary; 4 Syreon Research Africa, Accra, Ghana; 5 Medication Adherence Research Group, Center for Health Technology Assessment and Pharmacoeconomic Research, University of Pécs, Pécs, Hungary; 6 Department of Clinical Pharmacy and Pharmacology, Medication Adherence Expertise Center of the Northern Netherlands (MAECON), University Medical Center Groningen, University of Groningen, Groningen, Netherlands

**Keywords:** medication adherence, economic outcomes, clinical impact, burden, cost-effectiveness, chronic diseases, adherence, clinical outcomes

The funder Abbott Pharmaceuticals Ltd., was erroneously omitted. The correct **Funding** statement reads:

The author(s) declare that financial support was received for the research and/or publication of this article. The journal publication fee for this article was paid by Abbott Pharmaceuticals Ltd. The funder was not involved in the study design, collection, analysis, interpretation of data, the writing of this article, or the decision to submit it for publication.

The original article has been updated.

