# Supplementary material for: Clinical and economic consequences of medication nonadherence: a review of systematic reviews
Source: Front Pharmacol. 2025 Jun 25;16:1570359. doi: 10.3389/fphar.2025.1570359 (PMC12237677; doi:10.3389/fphar.2025.1570359)
Supplement: Supplementary file 1 [file Table1.docx]

**Supplementary materials**

Detailed search strings

| **Search**  **number** | **Search area** | **Pubmed** |
| --- | --- | --- |
|  |  | **Search terms** |
| #1 | Medication adherence | ("medication adherence"[MeSH Terms] OR "patient compliance"[MeSH Terms] OR adheren*[Title] OR persisten*[Title] OR complian*[Title] OR nonadheren*[Title] OR nonpersisten*[Title] OR noncomplian*[Title] OR non-adheren*[Title] OR non-persisten*[Title] OR non-complian*[Title]) |
| #2 | Clinical impact | ((clinical[Title/Abstract] AND (burden[Title/Abstract] OR impact[Title/Abstract])) OR "disease progression"[Title/Abstract] OR "clinical outcome"[Title/Abstract] OR "clinical deterioration"[Title/Abstract] OR "hospitalization"[Title/Abstract] OR "readmission"[Title/Abstract] OR "emergency visit"[Title/Abstract] OR "health outcome"[Title/Abstract] OR "morbidity"[Title/Abstract] OR "mortality"[Title/Abstract] OR "quality of life"[Title/Abstract] OR "QoL"[Title/Abstract] OR "life quality"[Title/Abstract] OR "health-related quality of life"[Title/Abstract] OR "HRQoL"[Title/Abstract]) |
| #3 | Economic impact | (((cost[Title/Abstract] OR resource[Title/Abstract] OR economic[Title/Abstract]) AND (burden[Title/Abstract] OR impact[Title/Abstract])) OR "out of pocket"[Title/Abstract] OR "co-payment"[Title/Abstract] OR copayment[Title/Abstract] OR "caregiver cost"[Title/Abstract] OR "caregiver expenditure"[Title/Abstract] OR "caregiver burden"[Title/Abstract] OR "carer burden"[Title/Abstract] OR "family burden"[Title/Abstract] OR "societal cost"[Title/Abstract] OR "social cost"[Title/Abstract] OR "work absenteeism"[Title/Abstract] OR "productivity loss"[Title/Abstract] OR "lost productivity"[Title/Abstract] OR "productivity impairment"[Title/Abstract]) |
| #4 | Combined searches | #1 AND #2 |
| #5 |  | #1 AND #3 |
| #6 | Combined searches limited to systematic reviews published in English | #4 Filters: English; Systematic Review |
| #7 |  | #5 Filters: English; Systematic Review |
| #8 | Combined searches limited to systematic reviews published in English after January 01, 2024 | #6 Filters: from 2014/1/1 - |
| #9 |  | #7 Filters: from 2014/1/1 - |
